# Using Instagram to Promote Youth Mental Health: Feasibility and Acceptability of a Brief Social Contact–Based Video Intervention to Reduce Depression Stigma

**DOI:** 10.1016/j.jaacop.2024.12.004

**Published:** 2025-02-06

**Authors:** Doron Amsalem, Andrés Martin, Lisa B. Dixon, Shilat Haim-Nachum

**Affiliations:** a New York State Psychiatric Institute, Columbia University Vagelos College of Physicians & Surgeons, New York, New York; b Child Study Center, Yale School of Medicine, New Haven, Connecticut; c Faculty of Medicine, Tel Aviv University, Tel Aviv, Israel; d School of Social Work, Tel Aviv University, Tel Aviv, Israel

**Keywords:** adolescents, depression, Instagram, social media, video

## Abstract

**Objective:**

Depression is a leading cause of disability among youth, with stigma significantly hindering mental health service utilization. Social media platforms offer a promising means to reach large, diverse youth populations, providing an opportunity to deliver interventions where young people are highly active. This study aimed to evaluate the feasibility and acceptability of delivering a proven social contact–based video intervention via Instagram. We hypothesized that the intervention would be feasible and acceptable, generating higher link clicks and lower cost-per-click compared with control videos.

**Method:**

A 2-week Instagram campaign in February 2024 targeted US adolescents ages 14 to 22. The campaign featured a 60-second human-narrated personal story video, previously tested and shown to reduce depression stigma. The intervention’s effectiveness was assessed using key metrics: impressions (the number of times the video was displayed), reach (the number of distinct viewers), link clicks (engagement with mental health resources), and cost-per-click (cost-effectiveness). These metrics were compared with 4 control videos that varied in narration style and content.

**Results:**

The campaign generated 808,000 impressions, reached 287,100 viewers, and resulted in 4,148 link clicks. The intervention video achieved 874 link clicks with a cost-per-click of $0.92, outperforming 3 of the 4 control videos.

**Conclusion:**

This study demonstrates that Instagram is a feasible and acceptable platform for disseminating evidence-based mental health interventions aimed at reducing depression stigma among youth. The findings support the potential for broader use of social media in public mental health strategies, though further research is needed to monitor subsequent help-seeking behaviors and assess impact across diverse groups.

**Clinical guidance:**

• Social media platforms like Instagram that have brief and visually engaging content may engage youth with mental health interventions.

• Incorporating social contact-based interventions, such as personal stories, may effectively reduce stigma and promote help-seeking behaviors.

• Pretesting mental health interventions in controlled settings could ensure content is both effective and safe before widespread dissemination.

• Target interventions toward age-appropriate audiences with culturally sensitive narratives to enhance relatability and engagement.

**Diversity & Inclusion Statement:**

We worked to ensure sex and gender balance in the recruitment of human participants. We worked to ensure race, ethnic, and/or other types of diversity in the recruitment of human participants. One or more of the authors of this paper self-identifies as a member of one or more historically underrepresented racial and/or ethnic groups in science. One or more of the authors of this paper self-identifies as a member of one or more historically underrepresented sexual and/or gender groups in science. We actively worked to promote sex and gender balance in our author group. We worked to ensure that the study questionnaires were prepared in an inclusive way.

## Plain language summary

This study demonstrates the feasibility and acceptability of using Instagram to deliver a brief social media-based video intervention to reduce depression stigma among US youth aged 14 to 22. A two-week campaign featured a 60-second human-narrated personal story video and generated over 808,000 impressions and 4,148 link clicks, with the intervention video outperforming most control videos in cost-effectiveness. These findings highlight Instagram's potential as a scalable platform for delivering evidence-based mental health interventions that engage youth and may promote help-seeking behaviors.

Depression is a leading cause of illness and disability in youth.^1^ Longer duration of untreated depression is associated with greater severity, poorer outcome, and cognitive impairment.^2^ In addition to structural barriers to service use, stigma toward mental health is a profound obstacle that interferes with seeking of needed services by individuals.^3^ Public stigma refers to negative attitudes that motivate people to fear, reject, and discriminate against individuals with mental illness, who may themselves internalize these stereotypes (self-stigma).^4^^,^^5^ Developmentally, youth (ages 14–22) are especially sensitive to stigma because of their stage of identity consolidation, characterized by strong needs for a sense of competence, social acceptance, and autonomy.^6^ Confronting public stigma and self-stigma early in life could prevent delays in treatment-seeking and reduce time to intervention.^7^ Reducing public sigma and self-stigma toward depression changes perceptions of treatment, a key factor in engaging youth with depressive symptoms in behavioral services.^8^ This study aimed to evaluate the feasibility and acceptability of delivering brief video interventions, previously proven to reduce depression stigma, via social media and promote help-seeking among youth.

Social contact–based interventions are one of the most effective ways to reduce stigma (public and self-sigma) and increase treatment-seeking.^9^ The core element of social contact–based programs is an empowered presenter with lived experience of a mental health condition who describes attaining their goals despite the illness.^10^ Social contact–based videos can reduce stigma toward depression and, in turn, increase treatment-seeking.^11^ Notably, the efficacy of video-based interventions is similar to that of in-person interventions^12^^,^^13^ and has numerous advantages, including lower cost, easier implementation, and broader dissemination; greater suitability for younger audiences, who may have shorter attention spans and prefer brief content due to rapid information consumption and frequent digital media usage^14^^,^^15^; and better alignment with current trends in social media usage, which is particularly relevant among adolescents.^16^

Social media plays a central role in the lives of young people, serving as a platform for both self-expression and communication. Its widespread use and strong influence among youth offer a valuable avenue for public mental health interventions.^17^ Platforms such as Instagram, known for their visually engaging and interactive content, have become essential components of social lives of youth. This underscores the importance of early intervention to prevent mental health issues from becoming long-term problems. However, these platforms can also perpetuate the stigma surrounding mental health, worsening the conditions and creating obstacles to seeking help.^18^ The challenge, therefore, is to leverage the positive aspects of social media to build resilience and provide support while minimizing the negative effects, such as misinformation and stigma, that can prevent young people from accessing crucial mental health services.^19^

Acknowledging the significant impact of social media on youth mental health, recent official reports and increasing public concern have highlighted the critical need to address these challenges.^16^^,^^20^ The White House Report on Mental Health Research Priorities^20^ and the Surgeon General’s Advisory^16^ both emphasized the importance of gaining a nuanced understanding of how social media influences the mental well-being of young people. These reports recognize the dual nature of social media, pointing out both its potential benefits and risks, and advocate for the development of targeted strategies aimed at protecting and promoting mental health among the younger population.

We previously used crowdsourcing platforms as a testing laboratory to iteratively assess the effectiveness of brief social contact–based video interventions aimed at reducing stigma and enhancing help-seeking intentions. This iterative approach enabled us to explore a broad spectrum of research questions, thereby deepening our understanding of potential moderators and mediators of the intervention effects. Specifically, we conducted a series of randomized controlled trials with 3,714 adolescents to determine whether brief social contact videos could reduce depression stigma and increase treatment-seeking intentions.^21^, ^22^, ^23^ We created 12 videos, including 6 intervention videos and 6 control videos. Each depression-related video featured an empowered young person sharing their personal story, describing their struggles with depressive symptoms; thoughts of life not being worth living; misconceptions about treatment; and how social support from family, friends, and professionals helped them overcome these challenges. The videos highlighted diverse protagonists, including Black, White, and transgender and gender-diverse individuals. Across studies, the depression videos significantly reduced stigma and increased treatment-seeking intentions among young viewers compared with the control videos.

The current study aimed to expand the outreach (ie, increasing visibility) and dissemination of evidence-based brief video interventions on Instagram to test youth engagement with the video and their help-seeking behaviors among adolescents ages 14 to 22 living in the United States. Specifically, we sought to test the feasibility and acceptability of a brief social contact–based video intervention compared with 4 control videos. We measured feasibility and acceptability using 2 metrics: impressions, which refers to the number of times the videos were displayed, and reach, the number of people who viewed the videos. Additionally, we aimed to compare link clicks, the number of participant clicks to the videos’ desired destination (ie, mental health help), and recruitment cost-per-click (CPC), calculated by dividing video costs by the total number of completed surveys across the 5 videos. CPCs, in practice, determine cost-effectiveness. These metrics were chosen based on prior research demonstrating their effectiveness in assessing engagement, feasibility, and acceptability of health interventions on social media.^24^, ^25^, ^26^, ^27^ We hypothesized that the intervention video would be feasible and acceptable to young Instagram users and that youth viewing the intervention video would exhibit higher link clicks and lower CPCs, indicating greater appeal and interest in the intervention video compared with youth viewing the control videos.

## Method

In February 2024, we launched a 2-week Instagram campaign featuring a 60-second video intervention previously proven effective in reducing depression stigma and increasing help-seeking intentions. This campaign included the intervention video and 4 control videos. We used sponsored, geographically targeted ads on Instagram to encourage participants—youth ages 14 to 22 currently living in the United States—to watch a short video. All video ads included the hashtags #depression and #fightingstigma, chosen based on a review of successful social media campaigns and aligned with the campaign’s focus on overcoming stigma and encouraging care-seeking.^28^^,^^29^ The ads also featured virtual links labeled “Learn more,” prompting viewers to seek mental health support. Instagram algorithms curated personalized content feeds, prioritizing individual interests, followers, posting frequency, and content timing. The video ads were displayed in a linear format, labeled as sponsored content within the user’s personal Instagram feed. The total cost of the experiment was $4,025, which is consistent with our standard budget for crowdsourcing experiments.

### Intervention and Control Videos

We conducted a focus group with Black girls diagnosed with depression to gain a better understanding of the stigma surrounding depression within this population.^22^ We then conducted a thematic analysis of the focus group discussions, adhering to established qualitative research methods, to develop a video script that accurately reflected the participants’ experiences.^22^ The script was subsequently reviewed and approved by the focus group participants to ensure it authentically represented their stories, and it was performed by a 16-year-old Black actress in the brief video, who described her experiences as a Black girl with depression. Involving professional actors along with appropriate disclaimers can mitigate the risk of exposure and safeguard individuals’ privacy, especially in the social media world where content can be easily shared widely. Our research supports this approach; in a study where young individuals with depression shared their personal stories of distressing experiences and recovery journeys through brief videos, either directly or via a professional actor, the outcomes demonstrated similar efficacy.^30^ This video has been repeatedly tested in several studies using a crowdsourcing laboratory, consistently showing efficacy in reducing depression stigma and increasing help-seeking, as described above.^21^^,^^22^

The control videos presented the same depression story either through an artificial intelligence (AI) bot (control 1) or via psychoeducational text that provided factual information about depression, including signs, symptoms, and help-seeking approaches, without including personal narratives. The AI-generated video in control 1 featured the same script as the intervention video, with similar race and gender characteristics of the protagonist, but was clearly presented by an AI bot rather than a human. Other videos were narrated by a human (control 2) or an AI bot (control 3) or presented without narration, accompanied by relaxing background music (control 4). This design was intended to test the hypothesis that personal narratives delivered by humans enhance viewer engagement and relatability by fostering identification and emotional connection, which was absent in the control 1 video. Controls 2, 3, and 4 specifically aimed to assess whether different presentation styles affect engagement levels and the effectiveness of conveying information about depression and reducing stigma. The inclusion of 4 different control videos was intended to reflect the variety of videos typically posted on Instagram, aligning with the study’s focus on acceptability and feasibility. Both the intervention and the 4 control videos were 60 seconds long. The video links are available in Supplement 1, available online.

We conducted A/B testing to compare the performance of a single intervention video with 4 control videos of equal length. A/B testing incorporates an embedded Meta Experiments tool allowing for the randomization of comparable datasets between the tested video ads while controlling for reach across different groups along with demographic elements (eg, gender). A/B testing helps to ensure participants will be evenly split and statistically comparable. Through A/B testing, it is possible to test how variable changes in social media algorithms perform to determine which one leads to better user engagement and satisfaction. For more details on this method, see Kohavi *et al.*^31^

## Results

Table 1 presents the definitions for impressions, reach, link clicks, and CPC. Regarding the feasibility and acceptability of brief social contact–based videos on social media, the experiment yielded 808,000 impressions (ie, the number of times the videos were displayed), reached 287,100 distinct viewers, and generated 4,148 link clicks (Table 2). The mean (SD) number of impressions across all videos was 161,600 (8,958), ranging from 148,000 to 174,000. The mean (SD) reach was 57,420 (2,352), ranging from 54,300 to 61,200. The total cost of the experiment was $4,025. The CI of the results was set at 95%.

**Table 1:** Outcome Measures

Term	Definition
Impressions	Number of times the videos were displayed
Reach	Number of people who viewed the videos
Link clicks	Number of participant clicks to desired destination of videos (eg, mental health help)
Cost-per-link	Video costs divided by total number of completed surveys across the 5 arms/videos

**Table 2:** A/B Testing of 5 Study Arms

Outcome	Intervention	Control 1	Control 2	Control 3	Control 4
Impressions, no.	167,000	174,000	163,000	148,000	156,000
Reach, no.	55,700	61,200	54,300	57,700	58,200
Amount spent, US dollar	$805	$805	$805	$805	$805

**Figure 1:**
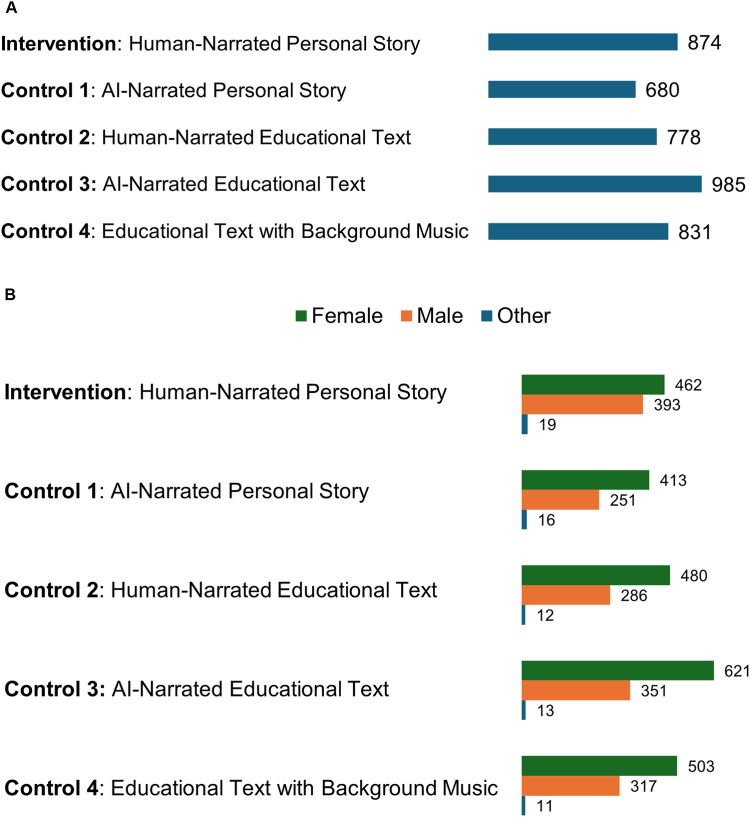
Link Clicks by Study Arm ***Note:****A, Link clicks per study arm. B, Link clicks per study arm by gender. AI = artificial intelligence.*

**Figure 2:**
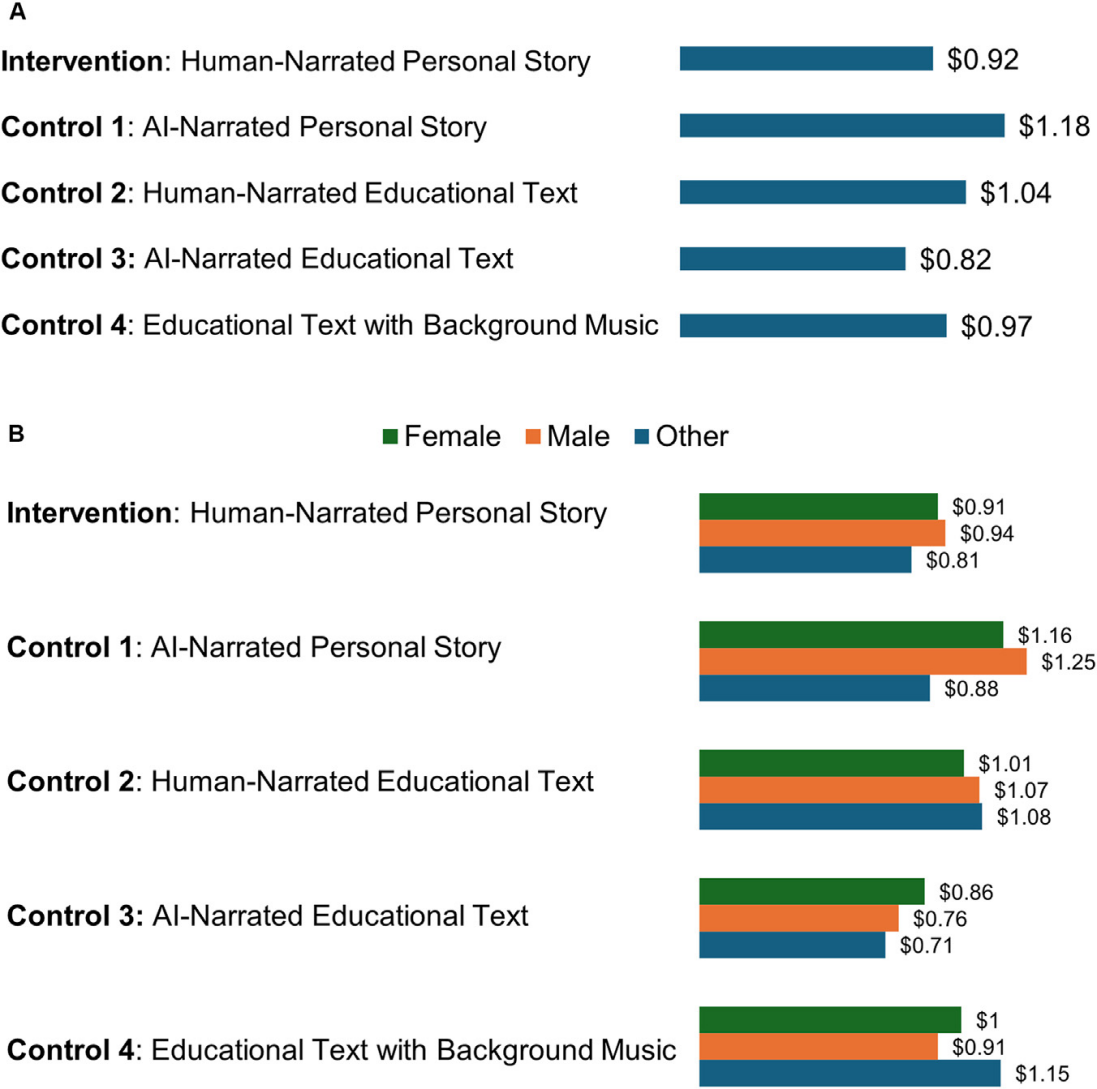
Cost Per Link Click ***Note:****A, Cost per link click per study arm. B, Cost per link click by gender. AI = artificial intelligence.*

Figure 1, Figure 2 present the comparison of the number of link clicks overall (Figure 1A) and by gender (Figure 1B) and the CPC overall (Figure 2A) and by gender (Figure 2B) for the intervention and the 4 control videos. The mean (SD) number of link clicks across all videos was 830 (108), ranging from 680 to 985, and the mean (SD) CPC was $0.99 ($0.12), ranging from $0.82 to $1.18. The human-narrated personal story (intervention) video performed strongly, achieving 874 link clicks and a CPC of $0.92, which was higher than 3 of the 4 control videos. The AI-narrated educational text (control 3) had the highest number of link clicks (985) and the lowest CPC ($0.82), whereas the AI-narrated personal story (control 1) had the fewest link clicks (680) and the highest CPC ($1.18). Overall, female and male youth showed similar patterns for link clicks and CPC.

## Discussion

This study sought to evaluate the feasibility and acceptability of delivering evidence-based brief video interventions to a large-scale audience of Instagram users, specifically targeting engagement and help-seeking behaviors among youth ages 14 to 22 in the United States. By comparing an intervention video with 4 control videos, we measured key outcomes, including impressions, reach, link clicks, and CPC, to determine the effectiveness and cost-efficiency of the intervention. The results demonstrated that all 5 videos successfully reached a broad audience, with a combined total of 808,000 impressions, 287,100 viewers, and 4,148 link clicks. The human-narrated personal story (intervention) video was highly effective, achieving 874 link clicks and a competitive CPC of $0.92, outperforming 3 of the 4 control videos. Overall, the findings suggest that the intervention video successfully engaged the target audience, supporting its feasibility and acceptability for broader dissemination. To our knowledge, this is the first study to disseminate evidence-based brief video interventions to reduce depression stigma among young Instagram users.

In line with previous reports,^16^^,^^20^ the principle of “first, do no harm” is paramount when considering the use of social media as a platform for mental health interventions. Whereas social media presents a valuable opportunity to reach a large-scale audience, it also presents potential significant risks to youth mental health. The personal story (intervention and control 1) and the psychoeducational text (controls 2-4) presented here were previously tested on crowdsourcing platforms that provided a controlled laboratory setting to rigorously test brief video interventions before they were disseminated in real-world social media environments.^9^, ^10^, ^11^ By pretesting content in these virtual laboratories, we can mitigate the risks associated with rolling out untested interventions on social media, safeguarding the well-being of young viewers. Similarly, Mheidly and Fares^18^ published the Infodemic Response Checklist to overcome the challenges posed by social media. Our team strongly recommends that all mental health interventions intended for social media be rigorously tested in controlled environments before being widely deployed to confirm that the content effectively reduces stigma and/or increases help-seeking behaviors, rather than having the opposite effect. This ensures that the interventions prioritize the safety and mental health of the audience.

The brief videos collectively yielded 808,000 impressions and reached 287,100 viewers, highlighting the effectiveness of these interventions. Notably, the study cost ($4,025) is comparable to the average cost of a crowdsourcing study,^32^^,^^33^ which typically reaches only a few thousand participants, yet our approach through social media achieved hundreds of thousands of impressions and reach. Although our study did not specifically target or screen for depressed youth, the high prevalence of depression in the population (approximately 20%) combined with the strategic use of depression-related hashtags and content preferences likely increased our reach to youth who are interested in depression-related content, know someone affected by depression, or are experiencing depression. The substantial reach and engagement metrics from our campaign support the likelihood that we successfully connected with youth experiencing depression or those experiencing it within their social circles.

A total of 4,148 viewers clicked on a link to learn more about mental health help, marking a significant first step in the help-seeking process. Although we did not measure whether these individuals actually sought help, the act of clicking the link suggests a level of engagement and intent that aligns with the early stages of behavior change models. A systematic review found that young people frequently seek help for mental health issues online due to benefits such as anonymity, ease of access, and control over the process.^34^ However, barriers such as low mental health literacy, privacy concerns, and doubts about the reliability of online resources persist. Other authors emphasized that mass media campaigns can reduce stigma and increase help-seeking behavior, but they must be carefully designed to avoid harmful effects.^35^ Whereas these findings are promising, they highlight the need for further research to explore the factors that influence whether individuals who express initial interest in mental health resources actually follow through with seeking professional help. Understanding these behaviors will be crucial in refining and improving the effectiveness of digital mental health interventions.

Some videos performed better than others. For example, the AI-narrated educational text (control 3) had the highest number of clicks (985) and the lowest CPC ($0.82), whereas the AI-narrated personal story (control 1) had the fewest clicks (680) and the highest CPC ($1.18). This variation suggests that factors such as narrative style and presentation method may significantly impact audience engagement. The fact that control 3 outperformed the intervention video in terms of clicks and cost-effectiveness may indicate that the straightforward, factual nature of the educational text, combined with the novelty of an AI narration, resonated more strongly with the audience than the personal narrative approach. It is possible that viewers may have perceived the AI-narrated educational text as more informative, timely, or credible. However, the scope of this study did not allow for a deep dive into the underlying reasons for these differences. Future research should focus on exploring these factors in greater detail, particularly to identify which elements of the interventions are most effective at driving engagement and promoting help-seeking behavior. By better understanding these dynamics, we can design more targeted and effective brief mental health video interventions that maximize engagement, lower costs, and ultimately lead to higher rates of help-seeking among young people in need.

This study has several limitations. First, whereas the intervention successfully engaged a large number of viewers, further research is needed to monitor and evaluate the actual help-seeking behaviors that followed this engagement. Second, due to the nature of this real-world campaign on social media, we were unable to gather comprehensive demographic data beyond age and gender. This limitation restricts our ability to analyze the impact of the intervention across different race and ethnic groups. Third, the reliance on self-reported data from social media platforms may introduce biases related to social desirability or self-selection. Additionally, Instagram’s analytics do not provide specific information on video watch durations or audience retention, limiting our understanding of engagement depth. Finally, the findings of this study are based on a single platform (Instagram), and the intervention video featured only 1 protagonist (Black), which may limit generalizability to other social media platforms and broader audiences. Future studies should include diverse protagonists and platforms to enhance applicability.

This study demonstrates the feasibility and acceptability of delivering evidence-based brief video interventions on Instagram to reduce depression stigma and promote help-seeking behaviors among youth. The results, with nearly a million impressions, substantial reach, and a high number of link clicks, suggest that Instagram may serve as an effective platform for delivering mental health interventions. However, it is crucial that such interventions are thoroughly tested, preferably in a controlled environment such as a crowdsourcing laboratory, to ensure that the content both is effective and resonates with the target audience before being widely disseminated on social media. Although the outcomes are promising, this pioneering study underscores the need for ongoing monitoring of actual help-seeking behaviors, the inclusion of diverse protagonists, and the collection of more comprehensive demographic data to enhance understanding and inform future research.

## CRediT authorship contribution statement

**Doron Amsalem:** Writing – original draft, Funding acquisition, Formal analysis, Conceptualization. **Andrés Martin:** Writing – review & editing. **Lisa B. Dixon:** Writing – review & editing. **Shilat Haim-Nachum:** Writing – review & editing, Formal analysis.
